# Pattern Recognition on Read Positioning in Next Generation Sequencing

**DOI:** 10.1371/journal.pone.0157033

**Published:** 2016-06-14

**Authors:** Boseon Byeon, Igor Kovalchuk

**Affiliations:** Department of Biological Sciences, University of Lethbridge, Lethbridge, Alberta, T1K 3M4, Canada; Tianjin University, CHINA

## Abstract

The usefulness and the utility of the next generation sequencing (NGS) technology are based on the assumption that the DNA or cDNA cleavage required to generate short sequence reads is random. Several previous reports suggest the existence of sequencing bias of NGS reads. To address this question in greater detail, we analyze NGS data from four organisms with different GC content, *Plasmodium falciparum* (19.39%), *Arabidopsis thaliana* (36.03%), *Homo sapiens* (40.91%) and *Streptomyces coelicolor* (72.00%). Using machine learning techniques, we recognize the pattern that the NGS read start is positioned in the local region where the nucleotide distribution is dissimilar from the global nucleotide distribution. We also demonstrate that the mono-nucleotide distribution underestimates sequencing bias, and the recognized pattern is explained largely by the distribution of multi-nucleotides (di-, tri-, and tetra- nucleotides) rather than mono-nucleotides. This implies that the correction of sequencing bias needs to be performed on the basis of the multi-nucleotide distribution. Providing companion software to quantify the effect of the recognized pattern on read positioning, we exemplify that the bias correction based on the mono-nucleotide distribution may not be sufficient to clean sequencing bias.

## Introduction

NGS is the most popular high-throughput sequencing technology in biological and medical research. DNA or cDNA fragment reads are mapped to a reference genome and the read enrichment is measured for experiments such as genome sequencing and transcriptome profiling [1,2]. The technology assumes that the DNA or cDNA cleavage is random and so the read start position is independent of the genomic sequence [3]. It therefore allows to use the number of reads mapping to certain regions of the genome as a quantitative measurement. Number of different techniques is used to reduce the size of DNA or cDNA molecules to accommodate them for sequencing that generate short reads. Shearing methods include physical methods, such as acoustic shearing, sonication and nebulization, chemical methods, such as use of high temperature and divalent metal cations, and enzymatic methods that include the use of restriction endonucleases or transposases. If, however, there is a bias in the pattern of DNA shearing that is dependent on certain DNA sequence context as well as the type of shearing used (or combination of sequence context and shearing method used), this bias may alter quantification of the results generated by NGS.

Previous reports indeed suggest that the sequencing bias around the read start position exists. Dohm et al. analyzed the sequences and number of the reads starting in a 1 kbp sliding window on the reference genome and found the positive correlation between the read frequency and the GC content [4]. Regions with elevated GC content contained increased number of reads, namely, windows with a GC content of 40% had almost twice as many reads as windows with 30% GC. In addition, analysis of sequencing errors in a particular sequence context revealed G nucleotide being the most frequent base immediately preceding erroneous base. Schwartz et al. examined the first twenty positions of all reads mapped to the reference genome and noticed the nucleotide biases at the beginning of reads, with more prominent bias observed in mRNA-seq (cDNA-seq) as compared to ChIP-seq (DNA-seq) data [5]. Benjamini et al. stratified the randomly sampled positions on the basis of the GC count in a sliding window, counted the number of read starts in the sampled locations, and derived the GC curve by estimating the mean read rates for each stratum [6]. They obtained the unimodal GC curve on which the read frequency increases with increasing GC in the low GC regions and the read frequency decreases with increasing GC in the high GC regions [6]. In addition, Poptsova et al. calculated the ratio between the numbers of nucleotides at the read start positions normalized by the average frequencies of the nucleotides in the ranges of -10 to -20 and +10 to +20 bp positions around the read start position, and observed the biases of nucleotides around the read start position [3].

Since the sequencing bias causes the improper under- or over-enrichment of reads over genomic regions, it may hamper the research effort leading to generation of false or misinterpreted data. Therefore it is demanded that the source of the sequencing bias is identified and its negative effect is reduced. It is therefore important that the pattern that decides the dependence between the read position and the genomic sequence is recognized. Previous work reports vague GC bias without specifying the magnitude to which the bias affects sequencing, and accomplishes the bias correction on the basis of G and C counts which may not be sufficient to clean sequencing bias [3–6].

We systemically recognize the pattern on read positioning and analyze its influence, using machine learning techniques. We define the classification problem to identify whether the read start exists or not at a given genomic position, and generate new features from sequences around read start positions over various read frequencies. Then we recognize the pattern that the NGS read start is positioned in the local region where the nucleotide distribution is dissimilar from the global nucleotide distribution, and quantify the effect of the recognized pattern on read positioning.

## Methods

### Illumina Sequencing data

Illumina NGS data of *Arabidopsis thaliana*, *Plasmodium falciparum*, Human, and *Streptomyces coelicolor* which contain various GC contents were downloaded (see Table 1). Also their reference genomes used for the specific data sets were downloaded from the data repositories indicated in the references. The raw sequencing file of *Streptomyces coelicolor* was mapped to the reference genome by Tophat with the option "-g 1" for no multiple alignment [7]. For data sets other than *Streptomyces coelicolor*, the downloaded bam files were used.

**Table 1:** Data types and sources.

Data	Type and mapped reads	Source
*Arabidopsis thaliana*	Illumina DNA sequencing data (The sequence data were produced by the DOE Joint Genome Institute). Mapped reads: 64610953	http://1001genomes.org/data/JGI/JGIHeazlewood2011/releases/2012_05_30/TAIR10/strains/Alc-0/Alc-0.bam
*Plasmodium falciparum*	Illumina RNA sequencing data. Mapped reads: 7586787	http://www.genedb.org/artemis/NAR/Malaria_RNASeq/version2.1.4/MAL_0h.bam
Human	Illumina DNA sequencing data. Mapped reads: 98428276	ftp://ftp.1000genomes.ebi.ac.uk/vol1/ftp/data/HG00106/alignment/HG00106.mapped.ILLUMINA.bwa.GBR.low_coverage.20120522.bam.csra
*Streptomyces coelicolor*	Illumina RNA sequencing data. Mapped reads: 26590790	http://www.ncbi.nlm.nih.gov/geo/query/acc.cgi?acc=GSM1378112

### Sequence data pools

**Fig 1:**
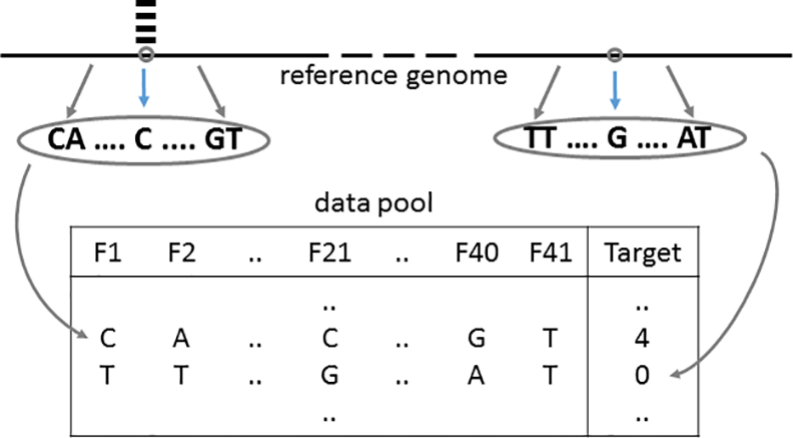
Generation of sequence data pool. Gray circles on reference genome indicate random positions, and black bars above reference genome indicate read starts at a given random position. Character “F” in data pool means feature.

Genomic positions were randomly selected and the frequencies of reads starting at the selected positions were extracted from data sets of four organisms mentioned above (see Table 2). Then data pools were created for each organism by using the reference sequence in the range of +/-20 bp from the selected positions as feature values and the frequency of reads starting at the selected position as target values (see Fig 1). Therefore, each data pool consists of 41 features, 1 target, and instances as many as the number of the selected positions. Note that the values of the first, 21^st^, and 41^st^ features of the data pool were the nucleotides at -20 bp from the selected position, at the selected position, and at the +20 bp from the selected position, respectively.

**Table 2:** Read start positions selected randomly. Chromosome numbers indicate the chromosomes used to create data pool, the number of positions is the number of positions selected randomly from the chromosomes, and the selected ratio is the ratio of the number of the randomly selected positions to the total number of positions on the chromosomes.

Read frequency	*Arabidopsis thaliana*		*Plasmodium falciparum*		Human		*Streptomyces coelicolor*	
	Chromosomes 1–5		Chromosomes 1–14		Chromosomes 1–22		Complete genome	
	Number of positions	Selected ratio	Number of positions	Selected ratio	Number of positions	Selected ratio	Number of positions	Selected ratio
0	5000	0.00	10000	0.00	2000	0.00	3000	0.00
1	500	0.00	1000	0.00	200	0.00	300	0.00
2	500	0.00	1000	0.00	200	0.00	300	0.00
3	500	0.00	1000	0.01	200	0.00	300	0.01
4	500	0.00	1000	0.01	200	0.02	300	0.01
5	500	0.01	1000	0.02	200	0.04	300	0.02
6	500	0.02	1000	0.03	200	0.05	300	0.03
7	500	0.04	1000	0.04	200	0.07	300	0.05
8	500	0.06	1000	0.06	200	0.08	300	0.07
9	500	0.09	1000	0.08	200	0.09	300	0.09
10	500	0.14	1000	0.10	200	0.11	300	0.11
11 or more	5000	0.08	10000	0.14	2000	0.15	3000	0.08

### Distance between global and local nucleotide distributions

Global nucleotide distribution was calculated by counting the mono- and di-nucleotides in all the genomic regions indicated in Table 2. Similarly, the local distributions were created by counting the mono- and di-nucleotides over various frequencies in the sequence data pools. Each mono-nucleotide was normalized by the total number of mono-nucleotides and each di-nucleotide by the total number of di-nucleotides. Then the Euclidean distance between the global and local nucleotide distributions was calculated as *√(∑*_*n*_ *(P1(n)–P2(n))*^*2*^*)*, where *√* is the square root, *n* indicates a nucleotide, and *P1(n)* and *P2(n)* are the local and global nucleotide distributions, respectively.

### Sequence data sets for classification

Sequence data sets for the binary classification of whether reads exist at a certain position or not were generated from the sequence data pools. To prevent classifiers from being biased to the majority class and thus generalized incompetently, balancing data between positions with and without reads was performed by sampling the equal number of instances from each class [8,9,10]. The instances with the target value of 0 in each sequence data pool were equally split into two sets of the train and test data and labeled as the class 0. Then the same number of instances with target value greater than 0, 5, and 10 were randomly selected from each sequence data pool, labeled the class 1, and added to the train and test sets without overlapping. Therefore, three train and three test sets were created for three classification problems to classify the positions with no read and with 1 or more reads, 6 or more reads, and 11 or more reads for each organism. Finally, the target values were replaced with the class labels. The class labels 0 and 1 indicate the states of the read existence in which the read either does not exist or does exist at the selected position, respectively. Therefore each classification data set consists of 41 features, 1 target, and instances as many as the number of the instances with the target value of 0 in the sequence data pool. Also, random data sets were generated by randomly shuffling the target values of each of train and test sets 10 times. The classification of the sequence data sets was performed by C4.5 decision tree and Bayesian network of Weka [11,12]. Those classifiers were employed for visualization of the pattern in the sequence data. Whereas other classifiers are too slow to be run on large size data or not applicable to non-numeric sequence data, or generate non-graphical outputs, decision tree and Bayesian network are relatively fast to run and allow simple graphical output [13,14,15].

### Feature extraction for pattern recognition

New features were extracted from the sequence data for classification. The local nucleotide distribution of each instance was calculated by counting the mono-, di-, tri-, and tetra-nucleotides in the instance and normalizing each mono-, di-, tri, and tetra-nucleotide count by the total number of mono-, di-, tri-, and tetra-nucleotides in the instance as follows: *(M*_*i*_ */ ∑*_*i = 1 to 4*_ *M*_*i*_*)*, *(D*_*j*_ */ ∑*_*j = 1 to 16*_ *D*_*j*_*)*, *(TR*_*k*_ */ ∑*_*k = 1 to 64*_ *TR*_*k*_*)*, *(TT*_*m*_ */ ∑*_*m = 1 to 256*_ *TT*_*m*_*)*, where *Mi*, *Dj*, *TRk*, and *TTm* indicate the counts of the *i*^*th*^ mono-, *j*^*th*^ di-, *k*^*th*^ tri-, and *m*^*th*^ tetra-nucleotides in the instance. The normalized counts in the local nucleotide distribution were used as the new feature values of the instance. Therefore new data sets which were composed of 4 mono-, 16 di-, 64 tri-, and 256 tetra-nucleotide distribution features and 1 target were generated for the pattern recognition on read positioning.

### Classification for the pattern recognition

*K*-nearest neighbor classifier was employed for classification of the data which were composed of the extracted features [16]. *K*-nearest neighbor chooses the *K* number of train instances nearest to a test instance, takes a vote with the class labels of the training instances chosen, and then assigns the majority class label to the test instance. The distance between instances can be calculated using Euclidean distance metric, *√(∑*_*x*_ *(F1(x)–F2(x))*^*2*^*)*, where *√* is the square root, *x* indicates a feature, and *F1(x)* and *F2(x)* are the feature values of two instances. The 3-NN classifier of Weka with the Euclidean distance metric was used as the implementation of K-nearest neighbor classifier [11,12].

The aim of classification was not to build a prediction model but to validate the distance-based observation that the distribution of positions with reads was significantly far from the distribution of positions with no reads in the Euclidean distance. The sophisticated classifiers such as the support vector machine and random forest or ensemble methods such as LibD3C sometimes achieve high accuracy in classification problems [11,8,17]. However, for validating the Euclidean distance-based observation, the classifiers that do not apply directly the Euclidean distance to classification are not useful, and ensemble methods to combine the outcomes of these multiple classifiers are not meaningful. Therefore, the *K*-nearest neighbor classifier directly applying the Euclidean distance to classification without changing data space was employed for the validation of the distance-based observation. Also data generated from different NGS experiments generally hold various degrees of sequencing bias since experiments are performed in the heterogeneous environment. Thus, the classification accuracy to express the magnitude of the pattern effect on read positioning was measured using the train and test sets generated from the same NGS experiment.

### Feature selection using genetic algorithm

When the feature selection methods in the filter approach were applied to the extracted feature data, *K*- nearest neighbor classifier achieved worse performance. Therefore, genetic algorithm was employed to select features relevant to the target concept for each data set. Genetic algorithm is a search and optimization algorithm which simulates the process of the natural selection in biology [18,19]. The population of feature sets is randomly generated in the initial generation, and then the optimal feature set is searched through the genetic operators of selection, crossover, and mutation in following generations [20]. The size of binary population was 50 and the number of generations was 20. The population values of 1 and 0 indicate that the corresponding feature is selected and not selected respectively. The probabilities of crossover and mutation were 0.8 and 0.05 respectively, and the elitism that the best individual in current generation replaced the worst individual in next generation was applied. The fitness value was the accuracy of test set classified by the 3-NN classifier trained on the corresponding train set. Since the aim of feature selection was not building a prediction model but identifying features important for the pattern recognized on the analyzed data, the test set was used in the fitness function. The algorithm was implemented in Java, importing the 3-NN classifier of Weka with Euclidean distance metric [11,12].

Feature selection with genetic algorithm is the wrapper approach that uses the induction algorithm to evaluate the selected subsets of features. Since the wrapper approach required substantial computation for data sets in large size, the running time of feature selection was measured with the processor of Intel Core i7 2.00GHz on Debian Linux 3.2.0-4-amd64 system.

### Quantification of pattern effect

A novel metric, pattern effect index (PEI) was defined as *((accuracy % of K-nearest neighbor classifier– 50) / 50)*. The PEI value 1 indicates that read positioning completely follows the pattern and the PEI value less than or equal to 0 means that read positioning is random. The mean and standard deviation of classification accuracies on random data sets related to Table 3 were 49.98% and 0.81, respectively. The 3 standard deviation from the mean accuracy is 52.41% of which the corresponding PEI value is 0.0482. Therefore, the PEI value greater than 0.05 was interpreted as read positioning affected by the pattern.

**Table 3:** Classification accuracies of indicated classifiers. Accuracies were averaged over four organisms. The number in parentheses is the standard deviation. 0 vs. 1 or more, 0 vs. 6 or more, and 0 vs. 11 or more indicate problems for classification of the positions with no read and with 1 or more reads, 6 or more reads, and 11 or more reads respectively. Random shuffling was performed 10 times for each data set.

Data set	Classifier	0 vs. 1 or more	0 vs. 6 or more	0 vs. 11 or more
Randomly shuffled data	C4.5	49.84% (0.82)	50.01% (0.67)	49.84% (0.78)
Randomly shuffled data	BN	50.08% (0.81)	50.00% (0.89)	50.14% (0.88)
Original data	C4.5	60.06% (3.90)	63.28% (6.21)	65.94% (6.00)
Original data	BN	63.53% (9.21)	65.49% (10.63)	66.98% (12.13)

Companion software to calculate PEI is provided at GitHub: https://github.com/PatternEffect/. The software was written using the R packages of Biostrings, class and gtools [21,22]. Because of the time-consuming procedure to extract features from sequence data, the software processes an individual chromosome instead of whole genome.

Before and after the correction of sequencing bias, PEIs were calculated by the companion software on the chromosome 1 of *Arabidopsis thaliana*. The bias correction was accomplished by correctGCbias of deepTools which removes reads from the GC-rich regions and adds reads to the AT-rich regions according to Benjamini’s method [6,23]. The effective genome size of deepTools was set to 85% of the *Arabidopsis thaliana* genome as indicated in [23].

### Complexity of sequences

The complexity of a sequence was calculated by TraMineR as *√((q(s)/q*_*max*_*)×(h(s)/h*_*max*_*))*, where *√* is the square root, *q(s)* the number of transitions in the sequence, *q*_*max*_ the maximum number of transitions, *h(s)* the within entropy, and *h*_*max*_ the theoretical maximum entropy [24]. The classification of the complexity data sets was performed by the 3-NN classifier of Weka with Euclidean distance metric [11,12]. For the classification based on the complexities of sequences, the data sets which were composed of 1 complexity feature and 1 target were generated.

## Results

### Classification of read start positions

In the classification problems to classify the states of the read existence, if read positioning holds some pattern, the classifiers trained on the train sets should be able to predict the class labels of the test sets. The existence of the pattern on read positioning was examined by C4.5 decision tree and Bayesian Network (BN).

After the class labels of the test set were predicted by the classifier which was trained on the corresponding train set, the percentages of the test instances classified correctly were measured as the classification accuracy. While the overall average and standard deviation of classification accuracies on the data sets with the randomly shuffled target values are 49.98% and 0.81 respectively (data not shown), the original data sets show the classification accuracies higher than 60% (see Table 3). On the original data sets, all classifiers achieve relatively lower accuracies in the classification problems of 0 vs. 1 or more reads, and perform better in the problems in which the positions with no read and with higher read frequency have to be classified.

The higher classification accuracies on the original data than the random data imply that there is some pattern on read positioning. However, during training process, C4.5 and BN generated complex tree and networks which used almost full features, and therefore no concrete knowledge of the pattern on read positioning was acquired from these tree and network.

### Global and local nucleotide distributions

**Fig 2:**
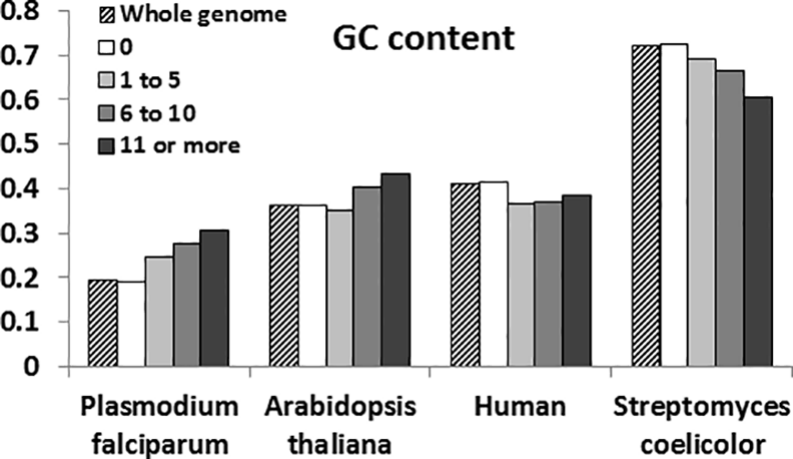
GC contents around the positions with various read frequencies. X-axis shows the organism analyzed and Y-axis displays the ratio of GC count to the total nucleotide count. Numbers of 0, 1 to 5, 6 to 10, and 11 or more indicate the read frequencies.

The GC contents around the position with no read are similar to the typically known GC contents, and the positions with high read frequency have GC contents that are higher or lower than typical GC contents in analyzed genomes (see Table 4 and Fig 2). Typical GC contents are 36.03% in *Arabidopsis thaliana*, 19.39% in *Plasmodium falciparum*, and 40.91% in human, 72.00% in *Streptomyces coelicolor* [25].

**Table 4:** GC contents around the positions with various read frequencies. Numbers of 0, 1 to 5, 6 to 10, and 11 or more indicate the read frequencies.

Data set	Whole genome	0	1 to 5	6 to 10	11 or more
*Arabidopsis thaliana*	36.03%	36.28%	35.30%	40.17%	43.24%
*Plasmodium falciparum*	19.39%	18.92%	24.46%	27.48%	30.54%
Human	40.91%	41.56%	36.60%	36.80%	38.38%
*Streptomyces coelicolor*	72.00%	72.47%	69.30%	66.44%	60.45%

**Fig 3:**
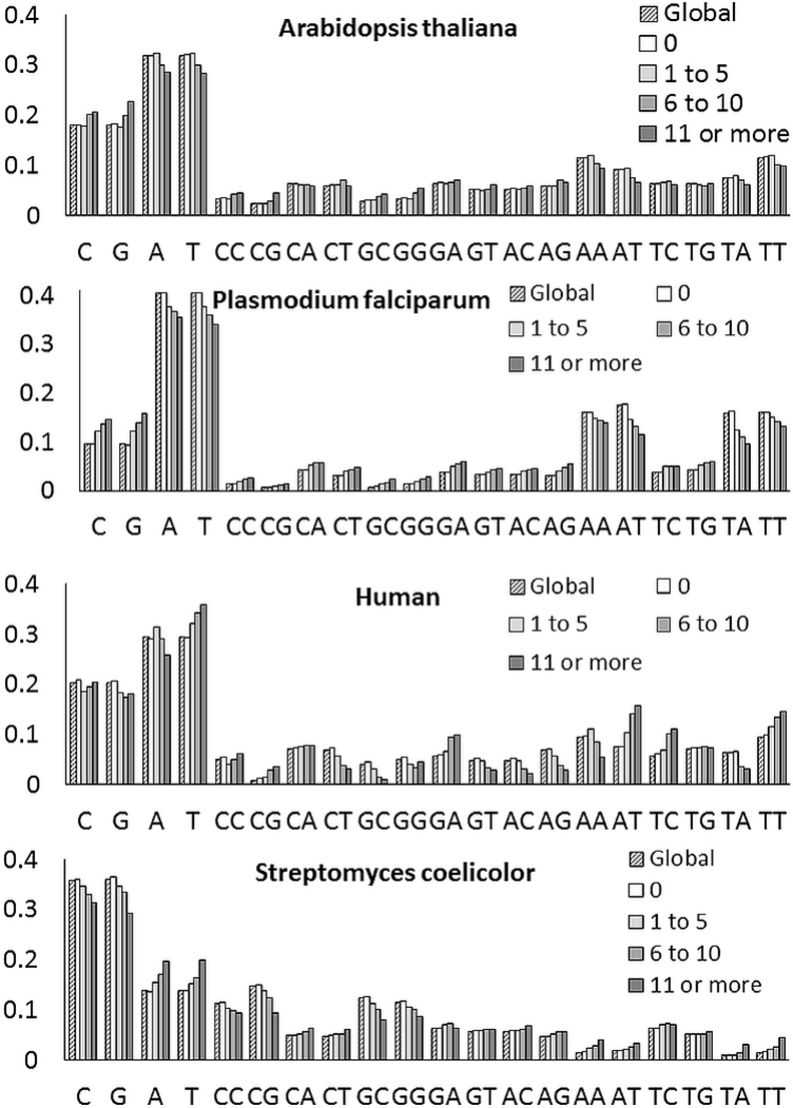
Global and local nucleotide distributions. X-axis shows mono- and dinucleotides and Y-axis displays the normalized nucleotide count. Numbers of 0, 1 to 5, 6 to 10, and 11 or more indicate the read frequencies.

Also, the local nucleotide distributions around the positions with no read are almost the same as the global nucleotide distribution, whereas the local distributions around the position with reads deviate from the global distribution (see Fig 3). In Euclidean distance, the nucleotide distribution of the positions with reads is significantly farther from the global distribution than the distribution of the positions with no read (see Table 5).

**Table 5:** Euclidean distances between the distribution of all local nucleotides and the global nucleotide distribution. Numbers of 0, 1 to 5, 6 to 10, and 11 or more indicate the read frequencies. The number in parentheses is the p-value of t-test, where the alternative hypothesis is that the nucleotide distribution of the positions with no read is closer to the global distribution than the distribution of the positions with reads.

Data set	0	1 to 5 (p = 0.0312)	6 to 10 (p = 0.0077)	11 or more (p = 0.0020)
*Arabidopsis thaliana*	0.0044	0.0118	0.0549	0.0921
*Plasmodium falciparum*	0.0074	0.0747	0.1157	0.1556
Human	0.0081	0.0632	0.1300	0.1683
*Streptomyces coelicolor*	0.0059	0.0381	0.0746	0.1503

### Pattern recognition on read positioning

It was observed that the nucleotide distribution around the position with reads is dissimilar to the nucleotide distribution around the position with no read, and the distributions are significantly far from each other in Euclidean distance. For validation of the distance-based observation, *K*-nearest neighbor classifier directly using Euclidean distance was employed to classify the instances with and without reads for the features representing the nucleotide distribution. The classification accuracies were measured for the three feature sets of mono-nucleotides, multi-nucleotides (di-, tri-, and tetra- nucleotides), and all nucleotides (mono- and multi-nucleotides).

*K*-nearest neighbor classifier on the data sets with the extracted features outperforms C4.5 decision tree and Bayesian Network on the original classification data sets (see Tables 3 and 6). In particular, the classifier achieves the accuracies higher than 70% for the multi-nucleotide and all nucleotide feature sets in the classification problems of 0 vs. 6 or more reads and 0 vs. 11 or more reads (see Table 6).

**Table 6:** Classification accuracies of *K*-nearest neighbor classifier and pattern effects. 0 vs. 1 or more, 0 vs. 6 or more, and 0 vs. 11 or more indicate problems for classification of the positions with no read and with 1 or more reads, 6 or more reads, and 11 or more reads respectively. Mono, multi, and all indicate the data sets which are composed of the features extracted from the distributions of mono-nucleotides, multi-nucleotides (di-, tri-, and tetra-nucleotides), and all nucleotides (mono- and multi-nucleotides), respectively. PEI was calculated from the classification accuracy for the feature set of all nucleotides and averaged over three classification problems.

Data set	0 vs. 1 or more			0 vs. 6 or more			0 vs. 11 or more			Average PEI
	Mono	Multi	All	Mono	Multi	All	Mono	Multi	All	
*Arabidopsis thaliana*	58.06%	70.84%	71.20%	62.56%	79.62%	79.50%	66.02%	89.12%	88.84%	0.60
*Plasmodium falciparum*	63.80%	67.70%	67.81%	67.24%	72.80%	72.84%	68.71%	78.11%	78.26%	0.46
Human	71.20%	82.55%	82.15%	74.85%	92.25%	92.20%	77.75%	93.75%	94.20%	0.79
*Streptomyces coelicolor*	65.10%	64.77%	65.37%	72.03%	73.90%	73.77%	77.03%	82.73%	82.87%	0.48

Given the observation on nucleotide distribution and the high classification accuracy of *K*-nearest neighbor classifier, we recognize the pattern that the read start is positioned in the local region where the nucleotide distribution is dissimilar from the global nucleotide distribution. The more deviated the local distribution is from the global distribution, the more reads are present in the region.

Interestingly, the classification accuracies for multi-nucleotide features are much higher than the accuracies for mono-nucleotide features and are similar to the accuracies for all nucleotide features (see Table 6). This implies that the mono-nucleotide distribution underestimates the pattern and the recognized pattern is explained largely by the multi-nucleotide distribution. For the identification of features important for the recognized pattern, genetic algorithm was also employed. The classification accuracies of classifiers using the features selected by genetic algorithm are shown in Table 7. Nine features were selected for all of the analyzed 12 data sets in the 3 classification problems for the 4 organisms, and the mono-nucleotides of A, C, G, and T were selected only for 2 or 3 data sets (see Table 8). This means that multi-nucleotides are more relevant to the explanation of recognized pattern than mono-nucleotides. Therefore, it is suggested that the correction of sequencing bias is performed on the basis of the multi-nucleotide distribution.

**Table 7:** Classification accuracies of *K*-nearest neighbor classifier for features selected by genetic algorithm. The value in parentheses indicate the running time in hour: minute.

Data set	0 vs. 1 or more	0 vs. 6 or more	0 vs. 11 or more
*Arabidopsis thaliana*	73.56% (16:09)	83.42% (16:11)	91.90% (16:49)
*Plasmodium falciparum*	69.92% (45:35)	74.70% (41:12)	79.01% (54:36)
Human	85.80% (02:41)	95.50% (02:29)	97.00% (02:25)
*Streptomyces coelicolor*	70.23% (05:45)	77.07% (05:39)	84.67% (05:32)

**Table 8:** Features selected by genetic algorithm and the number of data sets for which the features were selected. Features which were selected for 4 to 10 data sets are not shown.

Selected features	Number of data sets
TC, GAT, AAAA, CAAG, CGCG, CGGC, CGTA, GAAT, TCTT	12
AT, CA, GC, GT, TT, AAG, ACG, AGC, CCA, CCG, GGT, GTT, TTC, TTT, ACAC, ACAT, ACGT, AGCG, AGCT, AGGA, AGGG, ATCA, CACG, CAGG, CCAT, CCCT, CCTC, CGCA, CGGT, CGTC, CGTT, CTAA, CTAC, CTGA, CTGG, GAAA, GCAT, GGAT, GGCA, GTAC, GTTA, TATA, TCCC, TCGC, TGGC, TTGT, TTTG	11
A, C, T, CACC, GCTG, GTAA, TGGG	3
G, ATC	2

### Quantification of pattern effect on read positioning

PEI was calculated from the classification accuracies. According to PEIs in Table 6, read positioning in the analyzed data sets are affected substantially by the pattern recognized. Especially the PEI values for *Arabidopsis thaliana* and human are greater than 0.6, which means that the pattern dominates read positioning in the data sets.

The correction of sequencing bias for *Arabidopsis thaliana* was accomplished based on G and C counts, and PEIs of the chromosome 1 were calculated by the companion software (see Methods). Although PEIs for the corrected data are lower than those for the uncorrected data, read positioning is still affected by the recognized pattern (see Table 9). In particular, PEI in the classification problem of 0 vs. 11 or more reads is 0.64 after the correction. This suggests that the bias correction based on the mono-nucleotide distribution may not be sufficient to correct sequencing bias.

**Table 9:** Pattern effect index for chromosome 1 of *Arabidopsis thaliana*. Original and corrected data indicate that reads are uncorrected and GC-corrected, respectively. 0 vs. 1 or more, 0 vs. 6 or more, and 0 vs. 11 or more indicate problems for classification of the positions with no read and with 1 or more reads, 6 or more reads, and 11 or more reads, respectively. PEIs were measured 5 times by the companion software and averaged. The number is the average PEI and the number in parentheses is the standard deviation.

Data set	0 vs. 1 or more	0 vs. 6 or more	0 vs. 11 or more
Original data	0.22 (0.03)	0.43 (0.03)	0.73 (0.06)
Corrected data	0.08 (0.05)	0.14 (0.03)	0.64 (0.12)

## Discussion

NGS is the most popular sequencing technology, assuming that the read positions are random and independent of the genomic sequence. Sequencing bias around the read start position was previously reported [3–6]. Schwartz et al. (2011) [5] and Poptsova et al. (2014) [3] detected the nucleotide bias around read start positions regardless of read coverage, whereas Dohm et al. (2008) [4] observed the positive correlation between GC content and read coverage where the number of reads increased as GC content increased. According to our observation, while GC content is positively correlated to read coverage in the data sets of *Arabidopsis thaliana*, *Plasmodium falciparum*, and human, it is negatively correlated in the data set of *Streptomyces coelicolor*. Benjamini et al. (2012) analyzed the fragment rate as the continuous function of GC content and discovered the unimodal relation between the fragment rate and GC content, in which there was increasing coverage at the low GC content locations and decreasing coverage at the high GC content locations [6]. This approach is different from ours in that we analyzed GC contents as the discrete function of four read frequency groups.

The dependency of read coverage on sequence complexity was also previously reported [5]. The high sequence complexity may cause high coverage due to high mappability of reads. To explore the dependency, sequence complexities were calculated from the sequences of classification data and used as the feature to classify the read start positions. According to Tables 10 and 11, there exists a weak dependence between sequence complexity and read coverage and positions with higher read frequency are more predictable. However, the classification accuracies are relatively low.

**Table 10:** Sequence complexity. The numbers of 0, 1 to 5, 6 to 10, and 11 or more indicate the read frequencies. The numbers outside and inside parentheses are the average and standard deviation of sequence complexities, respectively. The complexities were calculated from the sequence data pools.

Data set	0	1 to 5	6 to 10	11 or more
*Arabidopsis thaliana*	0.65 (0.06)	0.65 (0.06)	0.67 (0.05)	0.67 (0.06)
*Plasmodium falciparum*	0.66 (0.10)	0.69 (0.08)	0.70 (0.07)	0.71 (0.07)
Human	0.69 (0.21)	0.74 (0.08)	0.75 (0.10)	0.74 (0.13)
*Streptomyces coelicolor*	0.81 (0.05)	0.83 (0.05)	0.83 (0.05)	0.84 (0.04)

**Table 11:** Classification accuracies of *K*-nearest neighbor classifier on the sequence complexity data.

Data set	0 vs. 1 or more	0 vs. 6 or more	0 vs. 11 or more
*Arabidopsis thaliana*	48.78%	52.22%	59.82%
*Plasmodium falciparum*	54.93%	55.20%	55.76%
Human	60.65%	62.00%	64.75%
*Streptomyces coelicolor*	56.87%	60.47%	62.73%

Fragmentation protocols can alter the quality of the sequencing runs by introducing bias if the fragmentation pattern is not random. In addition, the use of chemical or enzymatic methods can result in carryover of chemicals/enzymes into the library preparation, again influencing the read quality. Knierim et al. compared three fragmentation methods, namely, nebulization, sonication and enzymatic fragmentation and analyzed the quality scores at the 3′-ends of the fragments sequence using Roche 454 technology [26]. The authors did not find any significant differences in the quality scores obtained with the three different fragmentation methods.

Various fragmentation methods might introduce other types of DNA damage besides double strand breaks. Enzymatic fragmentation methods are prone to introduction of additional DNA damages, such as single strand breaks, resulting in deletions or point mutations, especially when insufficient amounts of ligase were added. Analysis of sequencing data performed by Knierim et al. did not show any significant difference of point mutation rates between the three fragmentation methods [26]. Also, indels were similarly frequent in sequences obtained after nebulization and sonication fragmentation methods, but occurred more often in sequences obtained after enzymatic fragmentation.

Analysis of ultra-short sequence reads generated by Illumina after DNA fragmentation by nebulization (*Helicobacter acinonychis*) or sonication (*Beta vulgaris*) did not reveal any sequence bias near the immediate vicinity of read start positions [4]; the authors concluded that fragmentation step generated random nucleotide fragments.

Benjamini et al. supposed that PCR was a source of the GC bias because sequences generated from a PCR-free protocol contained a reduced CG bias [6]. After investigating genomic sequences in diverse GC contents during the library preparation steps, Aird et al. confirmed that PCR indeed was a source of base-composition bias [27].

Processing and analyzing the large-scale NGS data is laborious and time-consuming. It is probable that a parallel computing framework, such as MapReduce, leads to the scalable and efficient performance in the alignment, mapping, and assembly of NGS sequences [28,29]. Further research of the parallel computing framework in processing and analyzing NGS sequence data is needed [29].

## Conclusions

We analyzed NGS data of four organisms with different GC contents using machine learning techniques. We found that the nucleotide distribution around the position with read was significantly dissimilar to the nucleotide distribution around the position with no read. Moreover, using K-nearest neighbor classifier measuring Euclidean distance between the nucleotide distributions around the positions with no read and with reads, we recognized the pattern that NGS read start was positioned in the local region where the nucleotide distribution was dissimilar from the global nucleotide distribution. It was observed that more deviation of the local distribution led to more reads positioned. Also providing the companion software to quantify the effect of the recognized pattern on NGS read positioning, we demonstrated that the mono-nucleotide distribution underestimated sequencing bias and the correction based on the mono-nucleotide distribution might not be sufficient to clean sequencing bias. Therefore we suggest that the bias correction is performed on the basis of the multi-nucleotide distribution.
